# Optimisation of GBFS, Fly Ash, and Nano-Silica Contents in Alkali-Activated Mortars

**DOI:** 10.3390/polym13162750

**Published:** 2021-08-16

**Authors:** Hassan Amer Algaifi, Abdeliazim Mustafa Mohamed, Eyad Alsuhaibani, Shahiron Shahidan, Fahed Alrshoudi, Ghasan Fahim Huseien, Suhaimi Abu Bakar

**Affiliations:** 1 Faculty of Civil Engineering and Built Environment, Universiti Tun Hussein Onn Malaysia, Parit Raja 86400, Malaysia; 2 Department of Civil Engineering, College of Engineering, Prince Sattam bin Abdulaziz University, Alkharj 11942, Saudi Arabia; a.bilal@psau.edu.sa; 3 Department of Civil Engineering, College of Engineering, Qassim University, Buraidah 51452, Saudi Arabia; e.alsuhaibani@qu.edu.sa; 4 Department of Civil Engineering, College of Engineering, King Saud University, Riyadh 11421, Saudi Arabia; falrshoudi@ksu.edu.sa; 5 Department of Building, School of Design and Environment, National University of Singapore, Singapore 117566, Singapore; 6 School of Civil Engineering, Faculty of Engineering, Universiti Teknologi Malaysia, Johor Bahru 81310, Malaysia; suhaimibakar@utm.my

**Keywords:** waste glass materials, alkali-activated mortars, optimisation, nano silica, fly ash, granulated blast-furnace slag, optimisation, mechanical properties

## Abstract

Although free-cement-based alkali-activated paste, mortar, and concrete have been recognised as sustainable and environmental-friendly materials, a considerable amount of effort is still being channeled to ascertain the best binary or ternary binders that would satisfy the requirements of strength and durability as well as environmental aspects. In this study, the mechanical properties of alkali-activated mortar (AAM) made with binary binders, involving fly ash (FA) and granulated blast-furnace slag (GBFS) as well as bottle glass waste nano-silica powder (BGWNP), were opti-mised using both experimentally and optimisation modelling through three scenarios. In the first scenario, the addition of BGWNP varied from 5% to 20%, while FA and GBFS were kept constant (30:70). In the second and third scenarios, BGWNP (5–20%) was added as the partial replacement of FA and GBFS, separately. The results show that the combination of binary binders (FA and GBFS) and BGWNP increased AAM’s strength compared to that of the control mixture for all scenarios. In addition, the findings also demonstrated that the replacement of FA by BGWNP was the most significant, while the effect of GBFS replacement by BGWNP was less significant. In particular, the highest improvement in compressive strength was recorded when FA, GBFS, and BGWNP were 61.6%, 30%, and 8.4%, respectively. Furthermore, the results of ANOVA (*p* values < 0.0001 and high F-values) as well as several statistical validation methods (*R* > 0.9, RAE < 0.1, RSE < 0.013, and RRSE < 0.116) confirmed that all the models were robust, reliable, and significant. Similarly, the data variation was found to be less than 5%, and the difference between the predicted *R*^2^ and adj. *R*^2^ was very small (<0.2), thus confirming that the proposed non-linear quadratic equations had the capability to predict for further observation. In conclusion, the use of BGWNP in AAM could act as a beneficial and sustainable strategy, not only to address environmental issues (e.g., landfill) but to also enhance strength properties.

## 1. Introduction

Green concrete has emerged as one of the main focuses within the research community, in which the subject is linked to the utilisation of agricultural [1,2,3,4], industrial [5,6], or by-product [7] waste materials as the replacement of cement or aggregate in both cement-based and free-cement-based materials involving paste, mortar, or concrete [8,9,10,11,12,13]. In particular, the topic of alkali-activated binders (AABs) has recently become highly favourable among academic civil engineers. This is because AABs are regarded as more sustainable and environmentally friendly materials compared to cement-based materials. In cement production, for instance, a huge quantity of carbon dioxide gas is released to the environment. In general, AABs are developed through the substitution of cement with alternative pozzolanic binders, such as metakaolin (MK), silica fume (SF), palm oil fuel ash (POFA), granulated blast-furnace slag (GBFS), and fly ash (FA) [14,15]. The variations in their chemical composition have since prompted researchers to utilise these pozzolanic binders not only to tackle current serious environmental issues, but also to achieve a high quality alkali-activated material. Huseien et al. [16] and Al-Majidi et al. [17] demonstrated that the utilisation of GBFS as a sole binder could improve the microstructure and durability of alkali-activated concrete, owing to its high calcium content. However, the high calcium availability in GBFS could impose negative impacts on the properties of alkali-activated materials, such as high plastic viscosity, rapid setting time, and high drying shrinkage [18]. Moreover, the resistance of GBFS-based alkali-activated materials to aggressive environments such as acid attacks is a concern. The utilisation of fly ash as an alternative binder has been extensively investigated and evaluated in the existing literature; however, several undesirable properties have also been observed in FA-based alkali-activated materials, including poor initial strength and high molarity of the alkaline activator solution. These findings prompted the use of nano-silica in FA-based alkali-activated concrete development by Nuaklong et al. [19], who used nano-silica made from rice husk ash to address the poor strength gain in the early stages. Natural pozzolan (NP) has also been used as the sole precursor to develop alkali-activated concrete [20]; however, the SiO_2_ content is lower than those of GBFS and FA. This fact prompted Ibrahim et al. [21] to improve the reaction kinetics through the partial replacement of NP with nano-silica (nSiO_2_). To the same end, many researchers have shifted their attention to utilising either binary or ternary binders to minimise the aforementioned limitations in the development of alkali-activated materials. For example, Robayo-Salazar et al. [22] evaluated the steel corrosion performance in alkali-activated concrete made with GBFS (30% by mass) and natural pozzolan (70% by mass). Recently, Faridmehr et al. [23] also used binary binders, involving both GBFS and FA, to develop alkali-activated concrete, which was aimed at obtaining an acceptable setting time and to avoid rapid strength gain. The results of their study demonstrated that the replacement of GBFS by FA led to a decrease in strength compared to that of the control mixture without FA. As such, it could be inferred that researchers are still struggling to obtain the best binder in terms of strength and durability as well as serious environmental issues, such as the excessive accumulation of waste materials. Comprehensive research is, therefore, still required to obtain the optimum value of nSiO_2_ made from waste bottle glass in alkali-activated materials, either as an addition to, or a replacement of, FA or GBFS. Such tasks in addition to variable interaction investigations are challenging via experimentation alone. As such, a robust optimisation tool is necessary to acquire the desired results [24]. Indeed, data optimisation could represent the best available solution to investigate the relationship among independent variables, as well as dependent variables, in a systematic way.

Accordingly, in recent years, numerous mathematical predictions and optimisation models have been developed within the research community, including artificial neural networks (ANNs) [25,26,27], metaheuristic algorithms [28,29], genetic expression programming (GEP) [30,31,32,33], adaptive neuro-fuzzy inference systems (ANFIS) [34,35,36], and response surface methodology (RSM) [37,38,39]. Among them, RSM is one of the best statistical techniques used for data optimization [40]. It is also useful for reducing the number of experiments required, as well as predicting accurate results [41]. For example, Hassan et al. [42] developed a RSM model to investigate the significant interaction between the nano and micro palm oil fuel ash (POFA) content, and the fresh and hardening properties of a high-strength concrete. The optimum values of nano and micro POFA were 1.5–2.85% and 10%, respectively, with the accuracy of the model proven using ANOVA and several statistical indicators. Similarly, Basri et al. [43] utilised RSM to determine the effect of four independent parameters on the flexural strength of a geopolymer. Shahmansouri et al. [44] also developed a RSM model to optimise the mechanical properties of GBFS-based geopolymers under different sodium hydroxide concentrations, GBFS to silica fume ratio, and GBFS to natural zeolite ratio. The optimum conditions for the first scenario were 6.19 M NaOH and 15.9% GBFS replacement with silica fume, while the second scenario was 30% GBFS replacement with natural zeolite and 5.28 M NaOH. The compressive strength of a geopolymer was also optimised with respect to fly ash and waste foundry sand compositions using a RSM model in another study reported by Venkatesan et al. [45]. The accuracy and significance of their model were verified using ANOVA, in which a high F-value and low *p*-value were obtained. In addition, the adjusted correlation of determination *R*^2^ value was more than 0.92, indicating the closeness of the predicted and experimental results. In the same context, Gao et al. [46] employed RSM to maximise the compressive strength of alkali-activated slag under different ratios of liquid to solid concentrations and alkali activator. Other predictions and optimisation models are also available in the present literature [47,48].

Based on the above, both experimental and theoretical guidelines are still in urgent demand to optimise the content of nano-silica made from glass bottle waste, FA, and GBFS, to maximise the compressive, tensile, and flexural strengths of alkali-activated material. This study, therefore, contributes to the body of knowledge by quickly and effectively providing the required information and assessing the significance and interaction between the involved reaction parameters. In addition, this present study will also promote the utilisation of glass waste materials as the partial replacement of binders, which will be not only feasible and beneficial for environmental issues but will also enhance its properties. Hence, an experimental work with three non-liner quadratic equations was undertaken to achieve the aim of this study. In addition, ANOVA and several statistical validation methods, such as relative absolute error (RAE), mean absolute error (MAE), relative standard error (RSE), root relative standard error (RRSE), and root mean square error (RMSE), were taken into account to assess the error variation between the predicted and experimental results. Moreover, the correlation between the experimental and predicted data was evaluated using a coefficient of determination (*R*^2^), correlation coefficient (*R*), adjusted *R*^2^, and predicted *R*^2^. Furthermore, microstructure tests such as FE-SEM, XRD, and XRF were also conducted to support the present findings.

## 2. Materials and Optimisation Modelling

### 2.1. Materials

**Figure 1:**
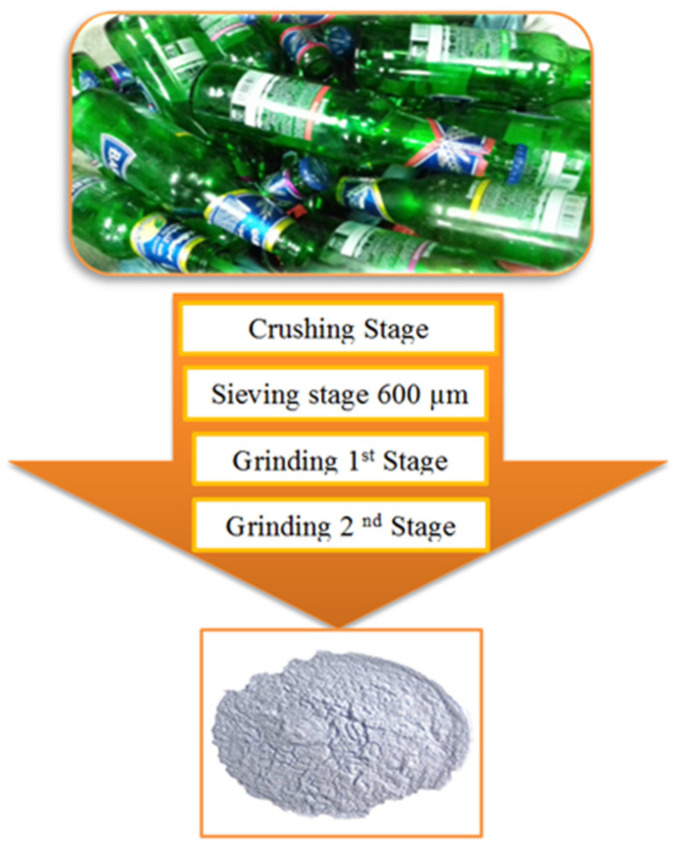
BGWNP production process.

In this present study, ternary blends of FA, GBFS, and BGWNP were utilised in the preparation of the alkali-activated mortars (AAM). All raw materials were collected from local waste industries in Malaysia. FA waste was collected from a Tanjung Bin power station in Johor. In addition, during the sample preparation process, the collected FA was used without any treatment as the main source of aluminium silicate. The pure free-cement binder (GBFS, from Iron industry) was acquired from Ipoh. It is important to note that the collected GBFS was composed of fine particles, with a size satisfying the specified requirement. As is well known, GBFS is considered as an excellent source of silica and calcium. Bottle glass waste was obtained from the local food and beverage industry in Johor. Several steps were taken to prepare the glass powder, as shown in Figure 1. In the first step, the glass bottles were washed using normal water, before being crushed with a crushing machine to achieve a finer material size of passing 600 µm. Following this, the sieved glass was ground for three hours using a Los Angeles abrasion machine of 25 kg capacity to produce particles of 25 μm in size. Subsequently, the fine glass was subjected to temperatures of 110 °C (±5) in an oven for 60 min. Finally, the glass powder was ground for a further 7 h using a bowl milling machine to achieve the optimum particle size (nano size).

Table 1 shows the chemical compositions of BGWNP, FA, and GBFS which were obtained from an X-ray fluorescence (XRF, HORIBA, Singapore) analysis. From the test results, primarily 86% of oxide elements were observed in FA, while 41.7% and 83% were related to GBFS and BGWNP, respectively. In addition, the calcium oxide content in GBFS (51.8%) was significantly higher compared to those of the FA and BGWNP samples. Indeed, the presence of calcium oxide, aluminium, and silicate play an important role in the synthesis of the alkali-activated material samples, particularly to activate the geopolymerisation process and ultimately form N,C-(A)-S-H gels. It should be noted that the level of potassium oxide (K_2_O) was found to be less than 1% in all three samples. Similarly, the sodium oxide (Na_2_O) content was 0.45%, 0.01%, and 0.08% for GBFS, BGWNP, and FA respectively. All three samples (BGWNP, FA, and GBFS) exhibited low loss on ignition (LOI) values. These positive results were consistent with ASTM C618 [49]. It is also interesting to note that the median particle size of the BGWNP sample was 80 nm, while those of the FA and GBFS samples were 10,000 nm and 12,800 nm, respectively. Furthermore, 100% of the BGWNP particle sizes passed 1 µm, while 100% of the GBFS and FA particle sizes were lower than 45 µm. It was observed that BGWNP was light-grey in colour, while the GBFS and FA particles were off-white and grey in colour, respectively

**Figure 2:**
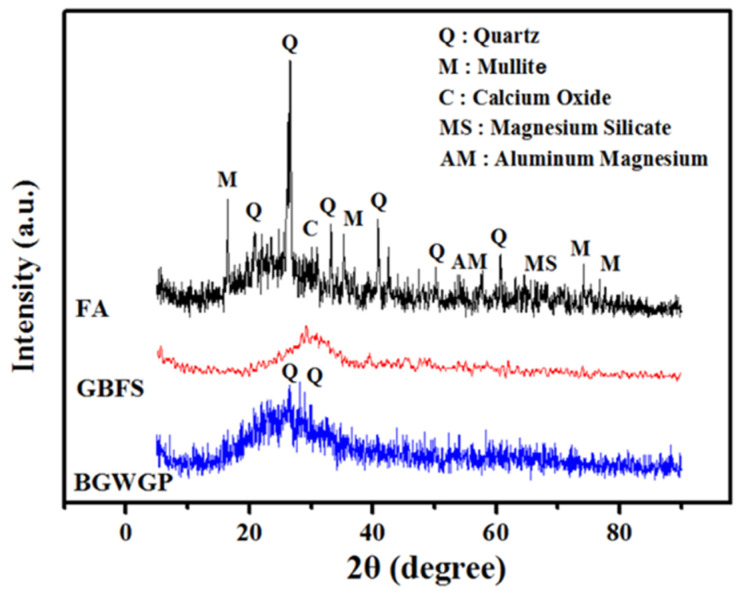
XRD patterns of FA, GBFS, and BGWNP.

An X-ray diffraction (XRD, Rigoku, Singapore) analysis was also conducted to chemically identify the end-products. Figure 2 presents the XRD analysis results of the three samples (BGWNP, FA, and GBFS). It could be seen that FA demonstrated significant diffraction peaks (2θ = 16–30°) owing to the presence of crystalline silica and alumina substances, with the remaining peaks presenting crystalline mullite and quartz phases. In addition, there was an absence of such notable peaks in the XRD pattern of GBFS, thus confirming the considerable amorphous characteristic of the sample. In particular, GBFS was distinguished by its high content of calcium and silica substances, which were necessary for the formation of GBFS. In addition, the high levels of reactive amorphous calcium and silica enable GBFS to effectively develop alkali-activated material. The XRD results also showed an amorphous characteristic in the case of BGWNP, indicating that the waste glass contained high levels of reactive aluminium and silica. The BGWNP sample was found to exhibit low-intensity crystalline quartz peaks at 2θ of 27° and 29°.

To prepare alkaline activator solution, both sodium hydroxide and sodium silicate were mixed to activate the aluminium silicate compound. Both the sodium silicate and sodium hydroxide (98%) used in this present study were supplied by QS, Malaysia. The analytical grade sodium silicate solution was comprised of H_2_O (55.80 wt.%), Na_2_O (14.70 wt.%), and SiO_2_ (29.5 wt.%). Meanwhile, the 2 M sodium hydroxide solution was diluted with normal water and left to cool at room temperature for 24 hrs. Subsequently, the sodium hydroxide solution was mixed with sodium silicate to prepare the alkaline-activated solution using a sodium silicate to sodium hydroxide ratio of 0.75. The final modulus of solution (Ms) of SiO_2_:Na_2_O was 1.02. To prepare the alkali-activated mortar samples, naturally occurring siliceous river sand was used as the fine aggregate. The collected sand was washed with water to remove silts and impurities, following the ASTM C117 standard [50]. Following this, an oven set at 60 °C was used to control the moisture content in the sand by exposure for 24 h. Finally, the river sand was sieved to ensure that the sand grade met the requirement of ASTM C33 [51].

### 2.2. Mix Design

In this present study, different mix proportions with various contents of FA, GBFS, and BGWNP were calculated according to ASTM C109 [52]. In particular, three mixes groups were adopted to evaluate the effect of nano-silica incorporation on the strength performance of alkali-activated mortar. For the first group, nano-silica was added to the FA and GBFS blend from 5% to 20% by weight. For the second group, nano-silica was used to partially replace the GBFS powder in the alkali-activated binder (5–20% by weight). For the last group, FA was partially replaced by nano-silica (5–20%). It should be noted that FA and GBFS were first blended at a ratio of 30% to 70% (30:70)as the control sample. In addition, the sodium hydroxide molarity (NH), modulus of solution (Ms), sodium silicate to sodium hydroxide ratio (NS: NH), alkaline solution to binder ratio (S:B), and binder to fine aggregate ratio (B:A) were kept constant at 2 M, 1.02, 0.75, 0.4, and 1 by mass weight for each level of replacement (see Table 2). To prepare the alkali-activated mortar, BGWNP, FA, and GBFS were first mixed for 3 min to ensure a uniform mixing. Then, the blend was further mixed with fine aggregate for 4 min. Subsequently, the activation process of the mixture was initiated by adding alkaline solution and mixing for an additional 5 min at medium velocity in a mixer machine. Tests were then carried out to ascertain the compressive, flexural, and splitting tensile strengths according to ASTM C579 [53], ASTM C78 [54], and ASTM C496 [55], respectively. In particular, the tensile strength of the mix was tested using a cylindrical mould (150 mm length, 75 mm diameter), while the flexural strength and compressive strength tests were carried out using a (40 × 40 × 160) mm prism mould and 50 mm^3^ cubical mould, respectively. The AAM samples were allowed to cure for a 24 h period in a controlled environment (24 ± 1.5 °C temperature, 75% relative humidity) prior to the demoulding process. In addition, to avoid any heat generation issues during the mixing process, the sodium silicate and sodium hydroxide solutions were mixed and cooled to the ambient temperature before they were used.

### 2.3. Microstructure and Morphology

To evaluate the effect of the BGWNP addition on the morphology and microstructure of AAM, three microstructure tests were adopted, including XRD, FESEM, and EDX. These tests were conducted on pulverised AAM powder, which was extracted from the core of each sample after 28 days of curing. The XRD analysis (JEOL, Singapore, Singapore), using Jade software, was used to identify the disordered phase of the AAM samples; where the test was conducted using a 2θ range of 3–55°, 0.02 step, and 0.5 s/step scanning speed. For the FESEM and EDX tests (Hitachi, Ibaraki, Japan), small portions of alkali-activated samples were collected from the strength test samples after 28 days. The samples were positioned on sample holders (brass stub type) using double-sided cellophane sheets and exposed to a five-minute period of drying by infrared radiation. Following this, the FESEM instrument was set at 20 kV and 1000× magnification to monitor the microstructure of the specimen. It should also be noted that the target samples were primarily gold coated using a sputter coating machine to increase the resolution of the microphotographs.

### 2.4. Optimisation Using RSM Model

Response surface methodology (RSM), which is recognised as a robust optimisation tool, has been extensively used in several previous studies [56,57,58]. It utilises statistical and mathematical approaches to accurately evaluate the significant interaction between the independent variables and outputs (dependent variables or responses). In this present study, RSM was adopted for the optimisation of the compressive strength (CS), tensile strength (TS), and flexural strength (FS) of the alkali-activated mortar incorporating binary binders (FA and GBFS) as well as BGWNP. To achieve the goal of this study, three models were developed and evaluated. In the first model, GBFS and FA content was maintained at 70% and 30%, while the amount of BGWNP (*X*_1_) was considered as the independent variable. In the second model, the replacement percentage of GBFS by BGWNP (*X*_2_) was considered as the independent variable, while the FA content was kept constant at 70%. In the third model, the replacement percentage of FA by BGWNP (*X*_3_) was considered as the independent variable, whereas the GBFS content was maintained at 30%. For all models, time (*X*_4_) was also considered as the independent variable. The minimum and maximum values of the independent variables are presented in Table 2. Design Expert software was utilised to develop the present models.

To mathematically obtain the relationship among the variables, a second-order polynomial equation was used, as described in Equation (1), where β_ii_ and β_ij_ referred to the quadratic and interactive coefficients, while the constant and linear coefficients were represented by β_o_ and β_i_, respectively.

(1) $$Y=\beta_{o}+\sum_{i}^{k}\beta_{i}X_{i}+\sum_{i}^{k}\beta_{ii}X_{i}^{2}+\sum_{ij}^{k}\beta_{ij}X_{ij}$$

For verification purpose, analysis of variance (ANOVA) was computed to evaluate the performance of the developed models. ANOVA is essential to investigate the significant interaction between the independent variables and responses. Indeed, numerous researchers have employed ANOVA in their previous studies [59,60,61,62]. In addition, the reliability and sensitivity of the models were also assessed using error statistical parameters as shown in Equations (2)–(7). In particular, relative absolute error (RAE), MAE, RSE, RRSE, mean square error (RMSE), and RRMSE were computed and evaluated, where *A*, *P*, $\bar{A}$, and *N* denoted the actual experimental data, predicted output of the RSM model, mean value of the actual data, and the number of experimental tests, respectively.

(2) $$RAE=\frac{\sum \left|\left(P-A\right)\right|}{\sum \left|\left(P-\frac{1}{N}\sum A\right)\right|}$$

(3) $$MAE=\frac{\sum \left|\left(P-A\right)\right|}{N}$$

(4) $$RSE=\frac{\sum {\left(P-A\right)}^{2}}{\sum {\left(P-\frac{1}{N}\sum A\right)}^{2}}$$

(5) $$RRSE=\sqrt{\frac{\sum {\left(P-A\right)}^{2}}{\sum {\left(P-\frac{1}{N}\sum A\right)}^{2}}}$$

(6) $$RMSE=\sqrt{\frac{1}{N}{\sum_{i=1}^{N}\left(P-A\right)}^{2}}$$

(7) $$RRMSE=\frac{\sqrt{\frac{1}{N}{\sum_{i=1}^{N}\left(P-A\right)}^{2}}}{\bar{A}}$$

Similarly, the correlations between the actual and obtained data from the RSM models were also examined using correlation statistical parameters as shown in Equations (8)–(10) [63,64]. Specifically, the determination coefficient (*R*^2^), correlation coefficient (*R*), and adjusted *R*^2^ were computed and evaluated, where *K* represented the number of independent parameters.

(8) $$R=\frac{{\left(N\sum PA-\sum A\sum P\right)}^{2}}{\left(N\sum A^{2}-{\left(\sum A\right)}^{2}\right)\left(N\sum P^{2}-{\left(\sum P\right)}^{2}\right)}$$

(9) $$R^{2}=\frac{\sum_{i=1}^{N}{\left(A-\bar{A}\right)}^{2}-\sum_{i=1}^{N}{\left(A-P\right)}^{2}}{\sum_{i=1}^{N}{\left(A-\bar{A}\right)}^{2}}$$

(10) $$R_{adj}^{2}=1-\left[\left(1-R^{2}\right)\times \frac{N-1}{N-K-1}\right]$$

## 3. Results

### 3.1. Predicted Equations and Their Validation

The mechanical properties of the alkali-activated mortars were predicted using three RSM models. These equations are useful to estimate and provide a quick insight into the evolution of the mechanical properties of the alkali-activated mortar made with a binary of FA and GBFS as well as nano silica. Table 3 shows the quadratic equations that represented the compressive, flexural, and tensile strength for all models.

**Figure 3:**
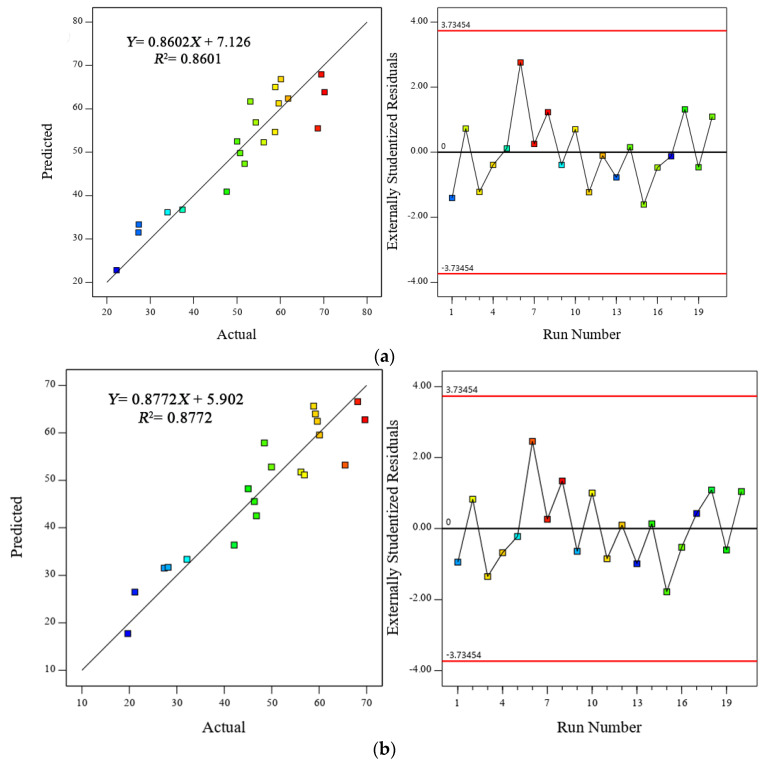

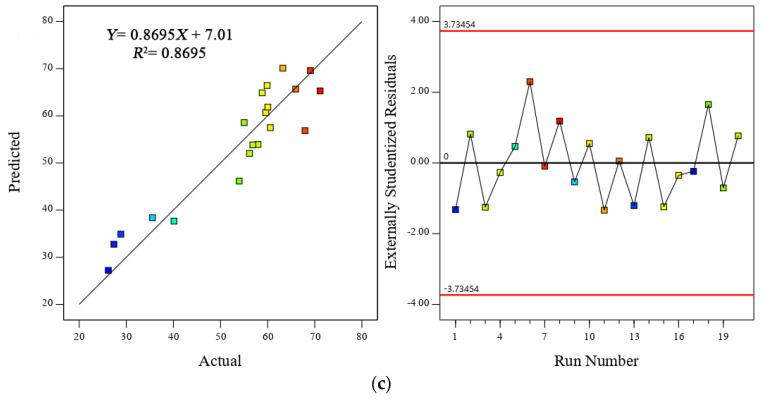
Correlation between the predicted and actual values of the compressive strength for (**a**) Model 1, (**b**) Model 2, and (**c**) Model 3.

The correlation and accuracy between the actual and predicted results of the compressive strength for all models were examined and evaluated. According to Mohammed et al. [65], a model is considered reasonable and accurate if the difference between the adjusted *R*^2^ and estimated *R*^2^ is small (<0.2). Similarly, Mohammed et al. [66] also stated that a good agreement and fitness could be obtained when the ratio between *R*^2^ and adjusted *R*^2^ is high (close to 1). In the present study, the difference between the adjusted *R*^2^ and estimated *R*^2^ was less than 0.08 for all data sets. In addition, the high ratio of *R*^2^ to adjusted *R*^2^ (approaching 1) indicated an excellent correlation between the predicted and experimental results. Moreover, an appropriate correlation was also obtained between the actual and predicted results using *R*^2^. For example, the values of the coefficient of determination, *R*^2^, of the compressive strength for Models 1, 2, and 3 were 0.8601, 0.8772, and 0.8695, respectively, as shown in Figure 3. This finding was consistent with Algaifi, Alqarni, Alyousef, Bakar, Ibrahim, Shahidan, Ibrahim and Salami [30] who stated that a strong correlation between the experimental and actual data is achieved when the *R*^2^ value is greater than 0.7. In the same context, the residual error values were found to be minimal and close to zero for all data sets. Moreover, the residual error was equally distributed across the whole range of the data, thus indicating that no error was present in the data.

The applicability of the proposed models was also investigated, as shown in Table 4. As is well known, the *p*-value is one of the most reliable statistical verification methods available in ANOVA, which is essential to validate the significance of regression coefficients. Indeed, a model term is considered significant when the *p*-value is less than 0.005, while a *p*-value of greater than 0.005 indicates that the model is not statistically significant. For the developed models of this present study, the *p*-values were found to be less than 0.005 for all terms, proving that all three models were significant. In addition, the F-value was also taken into account to evaluate the efficiency of the models. Typically, the F-value is used to assess the significance of the mean value variance, in which a high F-value indicates a significant model. In this present study, the F-values of all models were high, thus implying the robustness and reliability of the developed models for prediction. This was also in line with the findings reported in several previous studies. For example, in a study conducted by Ray, Haque, Ahmed and Nahin [38], the adequacy and significance of their model, which was used to predict the compressive and splitting tensile strength of concrete prepared with condensed milk can (tin) fibers (CMCF) and fine glass aggregate (GFA) were evaluated using F-value. In particular, the F-value was found to be high.

Moreover, several other statistical validation methods were also computed to assess and verify the performance of the proposed equations as shown in Table 5. According to Carrillo et al. [67], a strong correlation between the experimental data and predicted results is achieved when the *R* value is between 0.8 and 1, while a moderate correlation is obtained if the *R* value ranges between 0.5 and 0.8. This was also in line with another study reported by Khan et al. [68]. Moreover, close relationships between the experimental and estimated data have been proven with correlation values of 0.8–0.9 in many previous studies. In the present study, the correlation coefficients between the predicted and actual data of compressive strength for all models were 0.860, 0.877, and 0.870 respectively, confirming that the proposed models could be accurately used for prediction purposes. In the same context, the fitness of the models was also evaluated using RRSE. Ashrafian et al. [69] demonstrated that the value of RRSE should range between 0 and infinity. In addition, a perfect fit can be achieved when the RRSE value is zero. Farooq et al. [70] developed a model to predict the compressive strength of high performance concrete. Their model exhibited high performance with RSE values of 0.092 and 0.023 for both validation and test data respectively. As presented in Table 5, the RRSE values of the developed models of this present study were close to zero. Meanwhile, the RSE values of all models were of 0.012, 0.013, and 0.011 accordingly.

In the same regard, MAE and RMSE were also computed to verify the adequacy and efficiency of the models. These well-known parameters are also known as error indicators, and provide insight into the behaviour of the developed model. Generally, RMSE is more sensitive compared to MAE. This is because RMSE takes the square of the distance between the predicted and actual data into account [71]. Mohammed et al. [72] have previously proven the efficiency and capacity of several predictive models with small MAE values, specifically ranging between 3 and 11. Shah et al. [73] also proved the accuracy of compressive, flexural, and splitting tensile strength models using MAE and RMSE. In their findings, both MAE and RMSE values were considerably low. In particular, the MAE values ranged between 1.45 and 3.98, whereas the RMSE values varied from 2.43 to 3.92. In this study, as shown in Table 5, the MAE values of the proposed models were less than 4.4. Moreover, it was found that both RAE and RRMSE demonstrated minimum values (less than 0.1), thus confirming that the proposed models had the capability to predict with a minimum error. This was in line with a study by Shah et al. [74].

**Figure 4:**
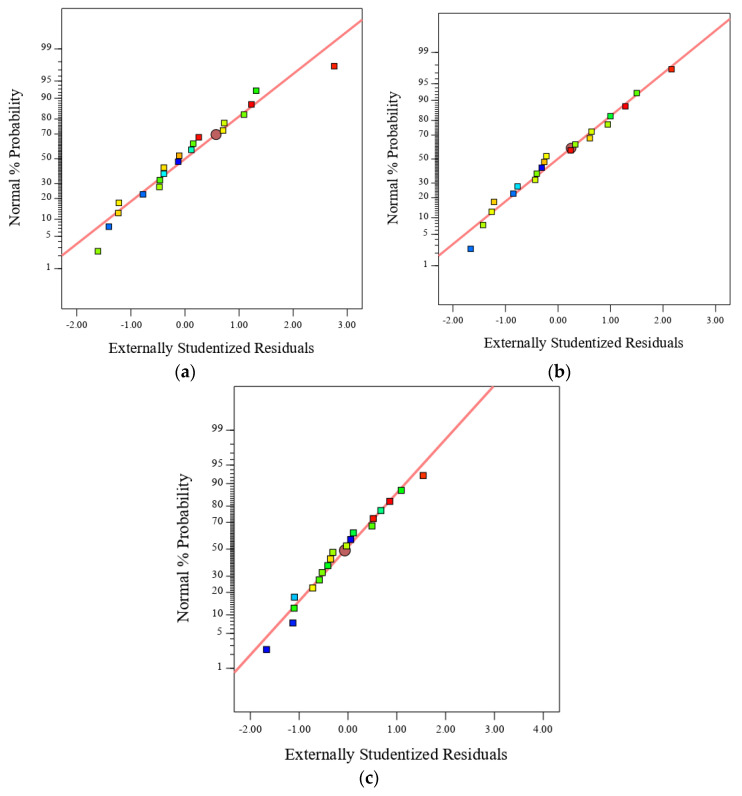
Normal probability plots of (**a**) compressive strength, (**b**) flexural strength, and (**c**) tensile strength.

In addition, the data distribution was evaluated using the normal probability method. A normal probability plot is widely used to assess data distribution. It could be seen from Figure 4 that all data were approximately normally distributed, as they were located and distributed along a straight line. This was in a good agreement with Ren et al. [75] who developed a RSM model to optimise the dosage of three different nano-materials in portal cement paste. Based on their study, the feasibility of their RSM model was proven, in which the measured residuals of compressive strength were normally distributed and depicted using a normal probability plot.

### 3.2. Interaction and Optimisation

As discussed earlier, the developed models for compressive, flexural, and tensile strength were verified using ANOVA and several statistical validation methods. As such, the next step was to obtain the optimum contents of FA, GBFS, and BGWNP. A multi-objective optimisation based on desirability functions was adopted to achieve this goal. This method is recognised as the best solution to determine the optimal values of the involved reaction parameters in the existing literature. The desirability function (*DR*) was expressed as in Equation (11), in which *n* represented the number of variables. In the present study, three dependent variables, namely compressive, flexural, and tensile strength were assessed, while four independent variables, namely FA, GBFS, and BGWNP contents, and time were used.

(11) $$DR={\left(d_{1}\times d_{2}\times d_{3}\times \ldots d_{n}\right)}^{\left(1/n\right)}$$

**Figure 5:**
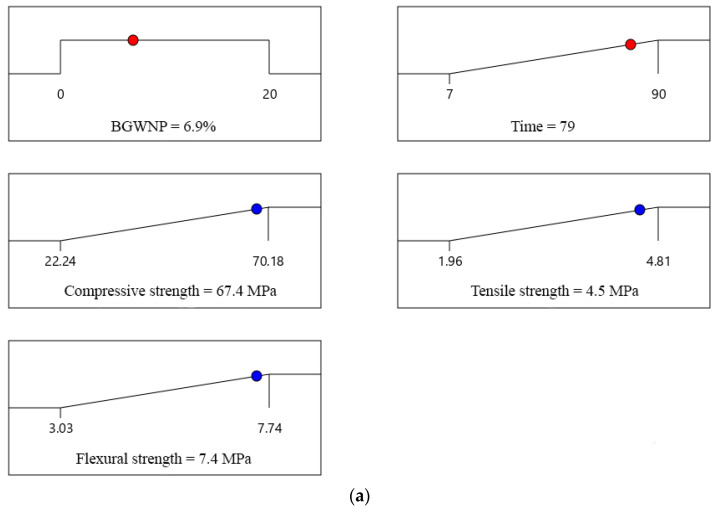

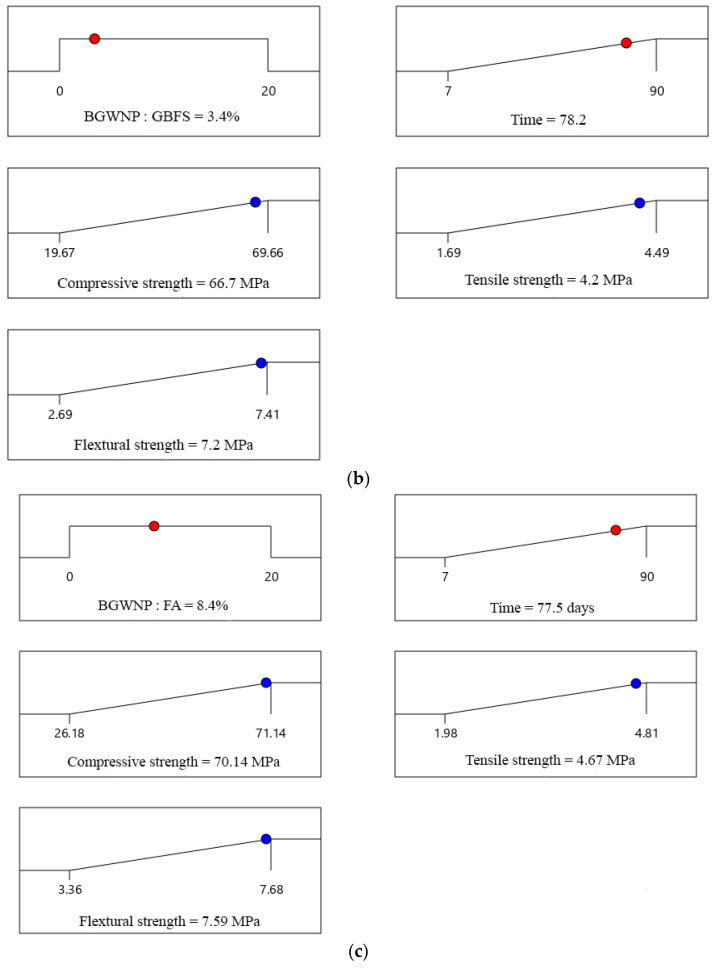
The optimum content of BGWNP (**a**) at constant FA-GBFS ratio (Model 1), (**b**) as the replacement of GBFS (Model 2), and (**c**) as the replacement of FA (Model 3).

It should be noted that three specific cases were considered for optimisation in this present study. In the first case, the goal was to obtain the optimum addition of BGWNP in FA-GBFS-based AAM. In particular, the ratio of FA to GBFS was kept constant (30:70) while BGWNP was defined as “in range”, as shown in Figure 5a. The goal for the second case was to determine the optimal replacement percentage of GBFS by BGWNP. In said case, the ratio of BGWNP to GFBS was defined as “in range” as shown in Figure 5b, while FA was kept constant (30%). The final case objective was to determine the optimal replacement percentage of FA by BGWNP. In this third case, the ratio of BGWNP to FA was defined as “in range” as shown in Figure 5c. Meanwhile, the compressive strength of AAM was defined as “maximum” for all cases.

Based on the results obtained, the greatest improvement in compressive strength of AAM was achieved when BGWNP of 8.4% was added as the replacement percentage of FA as shown in Figure 5c. In particular, the enhancement value of compressive strength was 18% compared to that of the control mix without the BGWNP addition (59.63 MPa). This finding confirmed that the replacement of FA by BGWNP was significant. The compressive strength slightly increased by 11% when BGWNP of 3.43% was added as the replacement of GBFS as shown in Figure 5b. A similar trend was also observed when BGWNP of 6.9% was added in FA-GBFS-based AAM as shown in Figure 5a.

**Figure 6:**
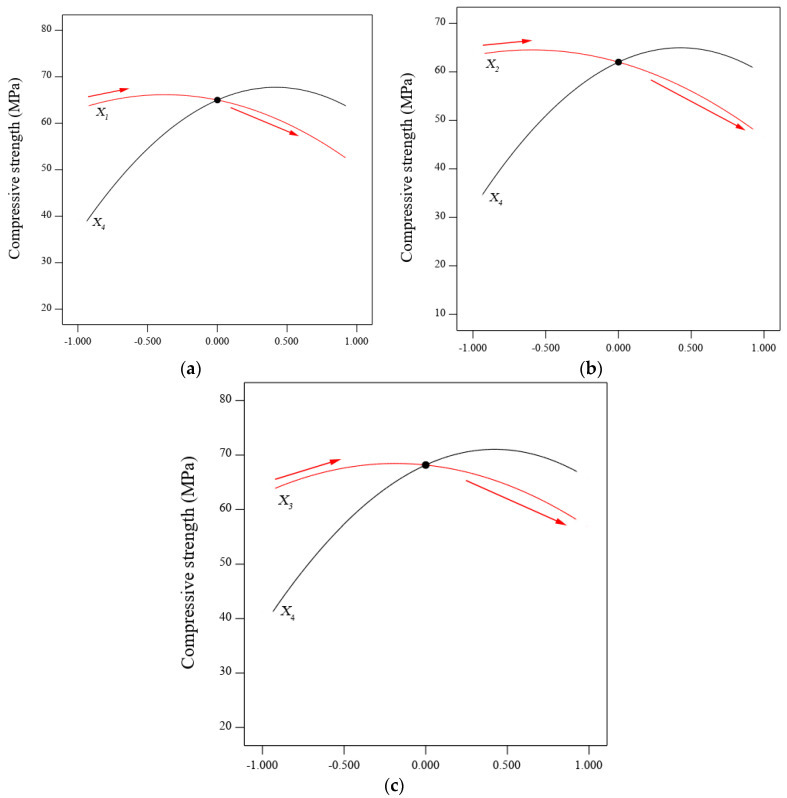
Interactions of BGWNP addition (**a**) at constant FA-GBFS ratio (Model 1), (**b**) as the replacement of GBFS (Model 2), and (**c**) as the replacement of FA (Model 3).

In the same context, the interaction and significance of the BGWNP addition based on the mechanical properties of the alkali-activated mortar was also investigated. It was observed that the improvement in compressive strength was the greatest with the BGWNP addition as a FA replacement as shown in Figure 6c. This was observed from the higher slope gradient of BGWNP compared to the other two scenarios, indicating a very strong interaction between the BGWNP addition as a FA replacement and compressive strength. In contrast, a very small slope gradient was observed for the replacement of GBFS with BGWNP, thus confirming an insignificant interaction with the BGWNP addition as a GBFS replacement, as shown in Figure 6b. Similarly, the compressive strength of AAM slightly increased with each increment of BGWNP content of up to 10%, as shown in Figure 6a. Beyond the 10% value, the strength gradually deceased, as illustrated for all cases. In general, it could be inferred that the interaction significance and sensitivity occurred in the sequence of (a) BGWNP addition as a FA replacement, followed by (b) BGWNP addition to AAM, and finally, (c) BGWNP addition as a GBFS replacement. This result was consistent with the previous mathematical findings (see Table 5), in which the *p*-value and F-value for the BGWNP addition as a FA replacement were higher than the others. This finding was similar to another study reported by Tian et al. [76] which investigated the significant interactions between the additions of NaOH, water, and sodium silicate, and fly ash to slag powder ratio, and the compressive strength of geopolymer. They disclosed that the *p*-values for the four linear variables were less than 0.5, indicating that all variables had a significant effect on the compressive strength of geopolymer. In addition, the significance ranking from low to high was: the sodium silicate addition, fly ash to slag ratio, the NaOH addition, and finally the water addition. In terms of strength evolution, the interaction significance ranking from high to low was: (a) the replacement of FA by BGWNP, (b) the addition of BGWNP in AAM, and finally, (c) the replacement of GBFS by BGWNP.

### 3.3. Effect of BGWNP Addition on the Mechanical Properties of FA-GBFS-Based AAM

**Figure 7:**
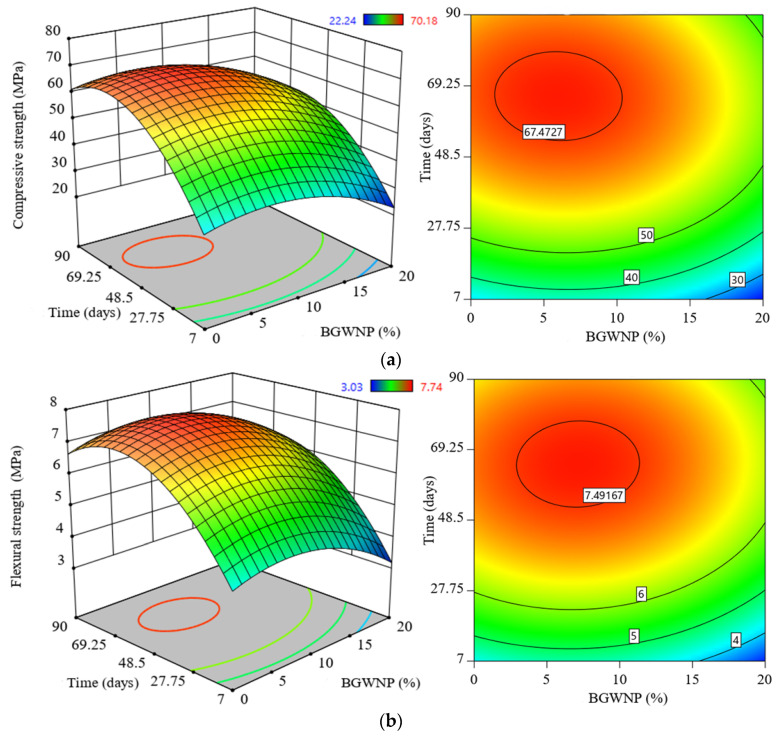

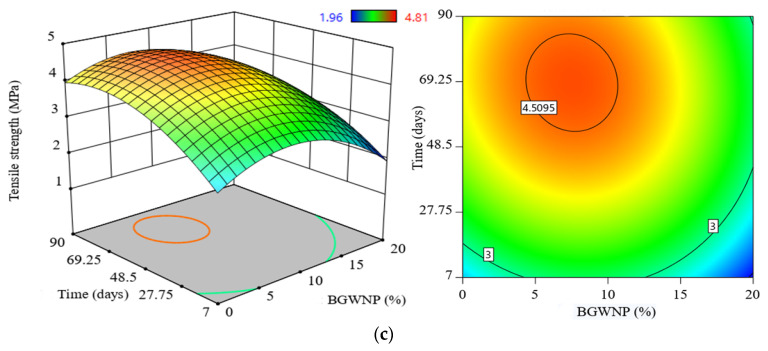
Evolution of (**a**) compressive strength, (**b**) flexural strength, and (**c**) tensile strength of FA-GBFS-based AAM with different amounts of BGWNP content.

**Figure 8:**
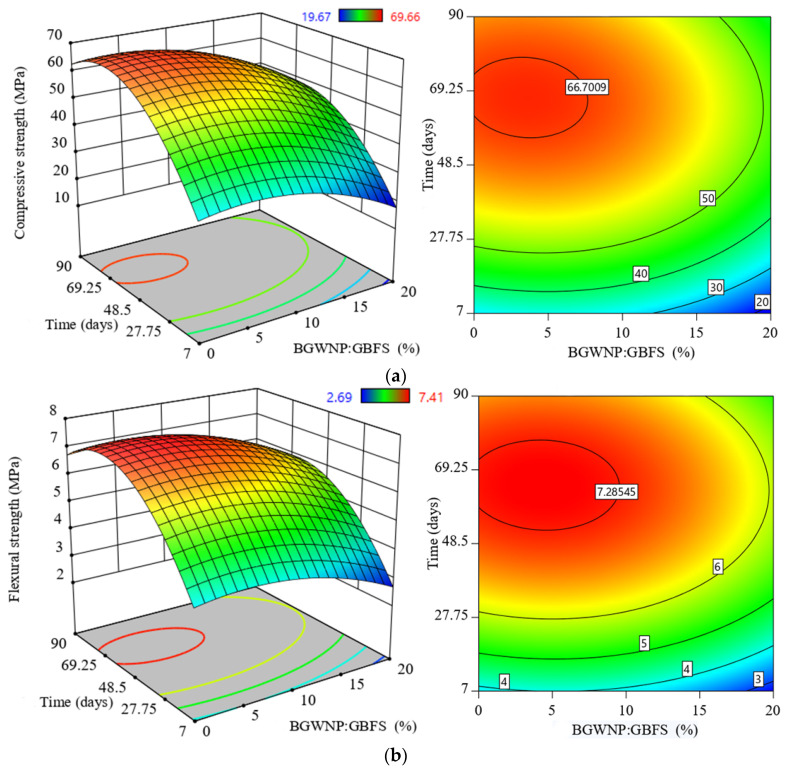

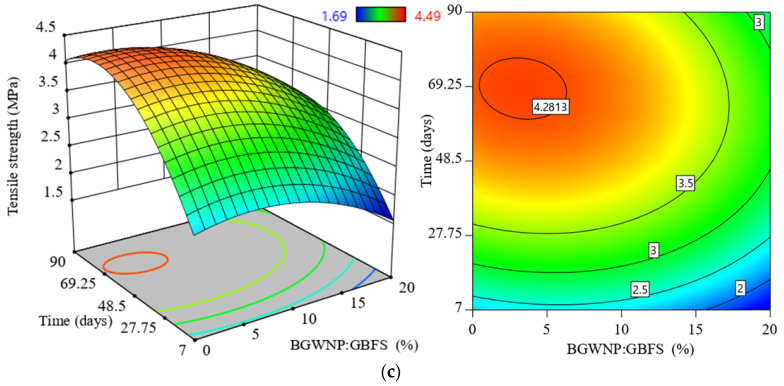
Evolution of (**a**) compressive strength, (**b**) flexural strength, and (**c**) tensile strength of FA-GBFS-based AAM with different replacement percentages of GBFS by BGWNP.

Figure 7 portrays the effect of the BGWNP addition on the strength performance of FA-GBFS-based AAM. In particular, three experimental parameters were considered to assess the effect of the BGWNP addition, involving compressive, tensile, and flexural strength. In general, it was found that the strength of all the alkali-activated mortar specimens increased with higher curing age of up to 28 days. Beyond 28 days, the increment of strength gains was not significant. Figure 7a presents the compressive strength evolution of the proposed AAM at various time intervals. It could be seen that the inclusion of BGWNP of up to 10% in an alkali-activated matrix had enhanced the compressive strength compared to that of the control matrix. In addition, the highest strength improvement was found to be 67.4 MPa with the addition of BGWNP (6.9%), in comparison with control mixture (59.6 MPa). In contrast, when the content of BGWNP increased up to 15 and 20%, the compressive strength decreased to 27.3 and 22.3 MPa, respectively. This was due to the greater water demand that had an adverse effect on the process of hydration. This was in good agreement with previous studies [77,78]. In the same context, as shown in Figure 7b and Figure 8c, the results indicated that the inclusion of BGWNP in the FA-GBFS binder had greatly enhanced the early flexural and tensile strength, as well as the later strength. For example, at day 78, the splitting tensile strength (4.5 MPa) and flexural strength (7.4 MPa) were achieved with the 6.9% addition of BGWNP, which was higher compared to those of the control samples of 3.7 MPa and 6.4 MPa.

### 3.4. Effect of BGWNP Addition as GBFS Replacement on the Mechanical Properties of FA-GBFS-Based AAM

The effect of the BGWNP addition as the partial replacement of GBFS on the AAM strength was also evaluated at several time intervals. As shown in Figure 8, as the curing time increased, the compressive, tensile, and flexural strengths of AAM also increased. However, the strength enhancement was not significant after 28 days. The strength improvement was achieved with the inclusion of BGWNP as a GBFS replacement of up to 5%. For example, improvement in the compressive strength was only observed with increments of BGWNP content of up to 5%. In particular, the compressive strength of AAM incorporating 3.4% BGWNP was 66.7 MPa after 78 days of curing, which was superior compared to that of the control mixture without BGWNP addition (59.6 MPa). Conversely, increasing the percentage of GBFS replacement by BGWNP beyond 5% negatively impacted the compressive strength, in which it dropped from 66.7 MPa to 48.3 MPa, and 45.1 MPa with 15, and 20% BGWNP content, respectively.

Similarly, the flexural strength of the AAM samples also exhibited improvement when the replacement of GBFS by BGWNP was set to no more than 5%, as shown in Figure 8b. For instance, it was observed that the flexural strength was 7.2 MPa when the replacement value was set at 3.4%, higher than that of the control mix without the BGWNP addition (6.3%). This positive result was linked to the enhanced AAM microstructure in the presence of nanoparticles (BGWNP). In contrast, a gradual reduction in the flexural strength of AAM was detected when the replacement percentage of GBFS by BGWNP was set to more than 10%. Specifically, the flexural strength of the AAM containing 15% BGWNP was 5.81 MPa, lower than that with a 3.4% BGWNP addition (7.2 MPa). Also, the flexural strength of the AMM incorporating 20% BGWNP was 5.4 MPa, which was lower than that of the AAM containing 15% BGWNP (5.81 MPa).

The results of the splitting tensile strength (STS) of AAM in the case of GBFS replacement by BGWNP are shown in Figure 8c. To aid comparison, the reference sample, which consisted of BGWNP and GBFS in a ratio of 5:20, was tested with a splitting tensile strength of 3.7 MPa. The highest value of STS was found to improve (4.2 MPa) when the BGWNP content was 3.4%. On the other hand, the AAM samples exhibited a loss of strength beyond a 10% BGWNP addition; of 2.9 and 2.8 MPa for AAM samples with BGWNP contents of 15 and 20%, respectively. It was also remarkable that the improvement in strength of AAM is attributed to the dispersion and reaction of BGWNP in the mortar matrix. As is well known, the pozzolanic reaction is considered as the sole parameter that affects pore size distribution, and thus strength evolution. For example, the compressive strength of the mortar steadily improved with an increasing nanoparticle content of up to 10%. Beyond this value, the strength was significantly reduced [79,80]. This finding complimented previous studies which found that the compressive strength of mortar was greatly enhanced when the addition of nanoparticles as a partial replacement of fly ash was set between 4% and 6% [81,82,83]. Such a strength improvement was attributed to the densified microstructure of the mortar matrix. This was also in line with other previous studies [77,78]. According to those studies, a reduction in strength was clearly noticeable when the nanoparticle content was set to higher than 10%. Similarly, the FS, STS, and MoE of AAM incorporating nanoparticles decreased when the nanoparticle addition level was higher than 10%. This was attributed to the decreasing level of calcium ions present in the mortar network [16].

### 3.5. Effect of BGWNP Addition as FA Replacement on the Mechanical Properties of FA-GBFS-Based AAM

**Figure 9:**
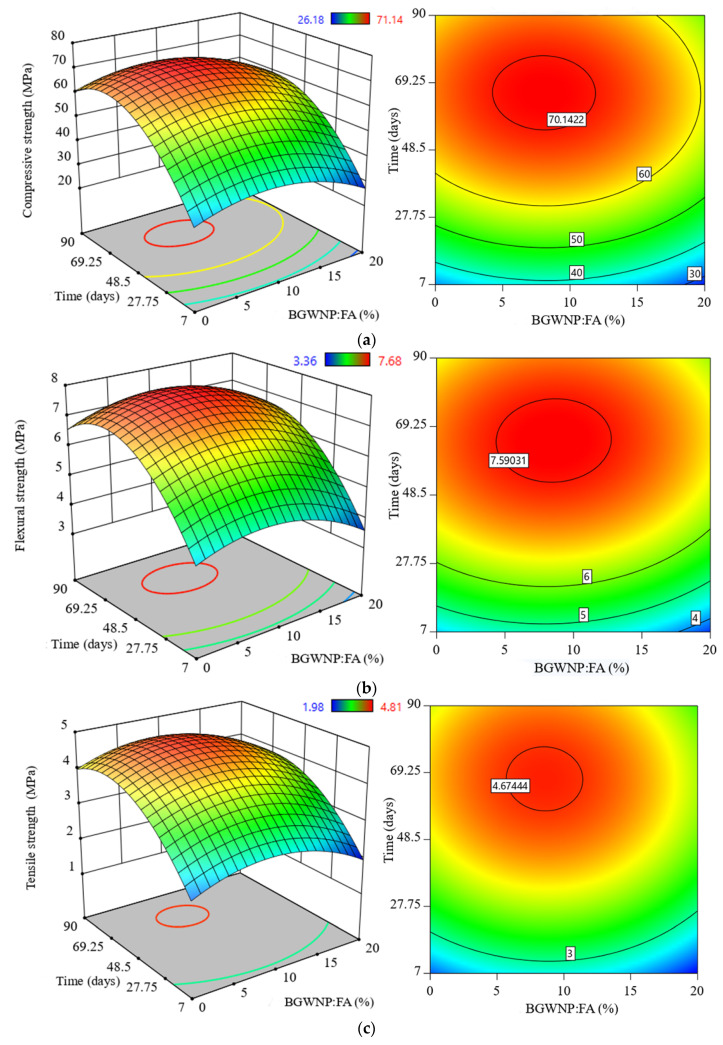
Evolution of (**a**) compressive strength, (**b**) flexural strength, and (**c**) tensile strength of FA-GBFS-based AAM with different replacement percentages of fly ash by BGWNP.

Figure 9 displays the effect of the BGWNP addition as a FA replacement on the compressive, flexural, and splitting tensile strength of AAM. In general, the results indicated that the strength of all specimens increased with higher curing age. However, the value of strength enhancement was not significant at later ages. Figure 9a presents the evolution of compressive strength of the AAM samples. It could be inferred that the greatest compressive strength was observed in the specimen containing 8.4% BGWNP with a recorded value of 70.14 MPa, which was superior to that of the control sample of 59.6 MPa after 78 days. Likewise, all the alkali-activated specimens which were prepared with BGWNP as a FA replacement exhibited enhancements in their flexural strength (Figure 9b) and splitting tensile strength (Figure 9c) with 8.4% replacement of FA by BGWNP at the early and later curing age. After 78 days of curing, the highest flexural strength (7.59 MPa) and tensile strength (4.67 MPa) were recorded in the specimen containing 8.4% BGWNP. As compared to the results achieved with the first and second groups, the greatest early and later strength were demonstrated by the specimen containing an acceptable amount of GBFS (30%) and 8.4% BGWNP addition as a FA replacement.

Nanoparticles are regarded as a nano ultra-filler material which play an important role in refining the microstructure of a cement-based material, and ultimately minimising the porosity of mortar. This fact was also confirmed by Lindgreen et al. [84] who demonstrated that nanoparticles tend to facilitate the filling of micro voids and pores inside the concrete microstructure. The modification or manipulation of the nanoparticles inside an alkali-activated material would render a new-fangled nanostructure [85,86,87]. It can be also noticed that the activity and efficiency of BGWNP in alkali-activated mortar might be comparable to the pozzolanic activity of FA (micro-silica) in term of strength, performance, and durability improvement [88,89,90]. This finding is consistent with Qing and Zenan [89] who stated that concrete incorporating nano-silica could gain higher early strength compared to that with silica fume (micro-silica). Moreover, it was observed that the workability of concrete containing nano-silica improved even with the decrement of superplasticisers content.

### 3.6. Microstructure

**Figure 10:**
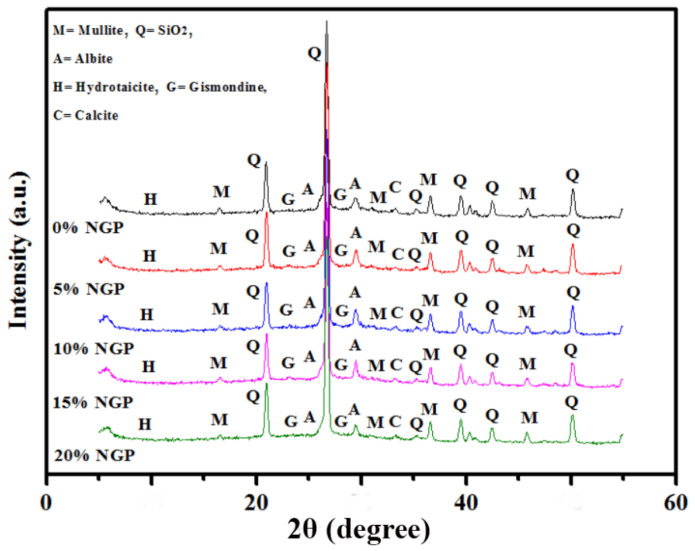
XRD patterns of AAM with various BGWNP contents.

The impact and activity of BGWNP at different percentages (0, 5, 10, 15, and 20%) inside the microstructure of alkali-activated mortar were also examined after 28 days of curing using XRD, FESEM, and EDX analyses. The XRD patterns of the AAM samples are illustrated in Figure 10. The presence of AAM gel was clearly observed, in which the XRD peaks were located between 20° and 35°. From the XRD patterns, the intensity of both gismondine (CaAl_2_Si_2_O_8_·4(H_2_O)) and albite (Na_0.95_Ca_0.05_Al_1.05_Si_2.95_O_8_) phases increased in the range between 24° and 34°. Meanwhile, a reduction in quartz (SiO_2_) intensity was observed at 36^o^ in the presence of BGWNP. The reduction in quartz peak intensity for gismondine and albite phases confirmed that more C,N-(A)-S-H gels were formed. This product is responsible for improving both hydration and geopolymerisation processes. In addition, the highest peaks of gismondine and albite were detected only in AAM incorporating 5% and 10% BGWNP. On the contrary, a limited production of C,N-(A)-S-H gels was observed with the replacement levels of BGWNP of 15% and 20%. This finding confirmed the previous predicted and actual results in which the strength of AAM decreased when the BGWNP addition level was set to more than 10%.

**Figure 11:**
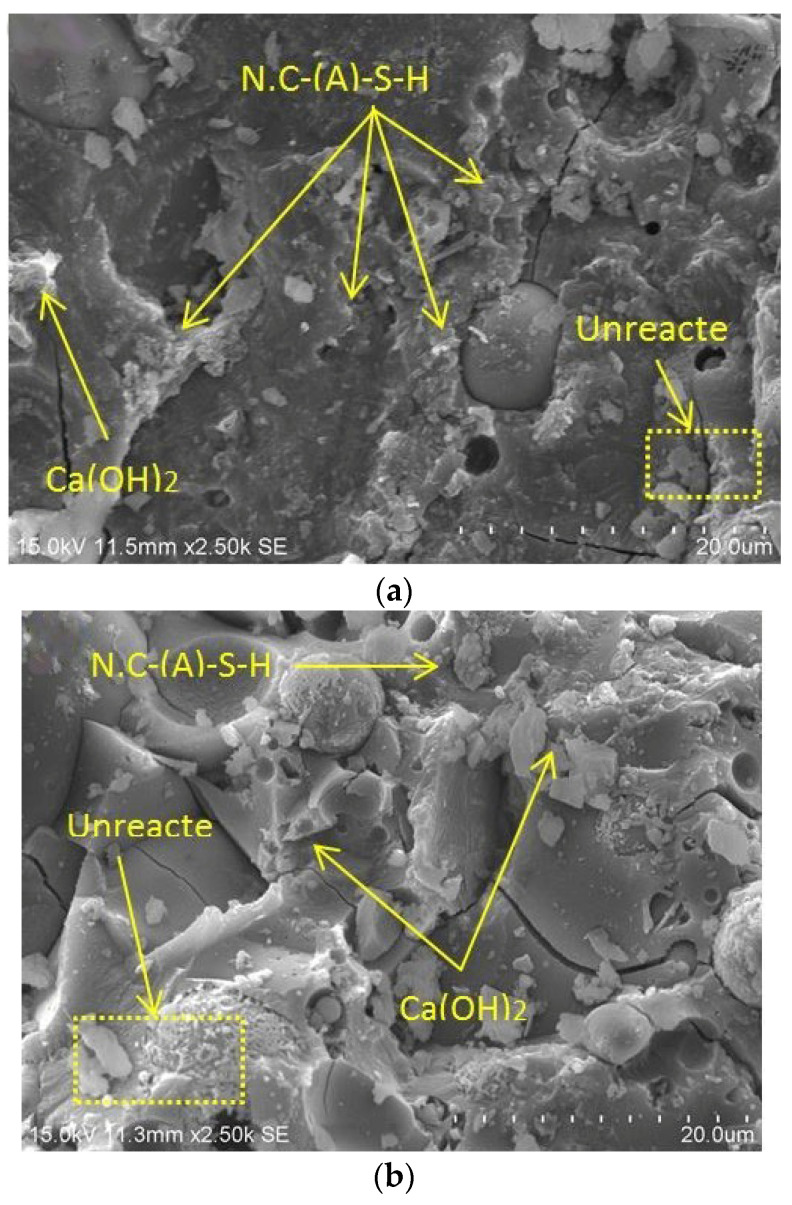
FESEM images of AAM containing various ratios of BGWNP (**a**) 5% and (**b**) 15% BGWNP.

**Figure 12:**
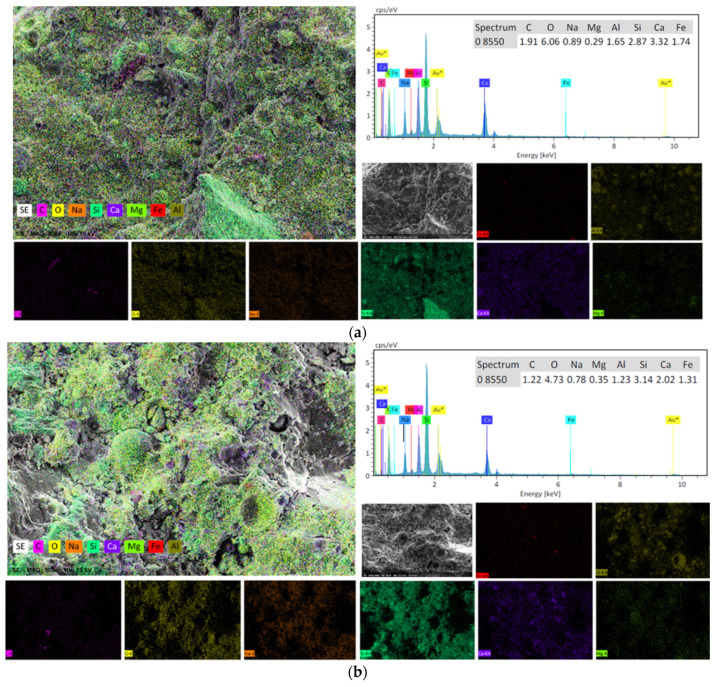
EDX images of AAM containing (**a**) 5% and (**b**) 15% BGWNP.

Similarly, both FESEM and EDX analyses were carried out to investigate the change in the microstructure of AAM due to the addition of BGWNP, as shown in Figure 11 and Figure 12, respectively. These tests were also intended to support both the predicted and experimental results. Based on Figure 11a,b, it could be seen that the structure of the AAM incorporating 5% and 10% BGWNP was dense with less non-reacted particles and pores. A partial reaction of fly ash particles was also observed, as shown in Figure 11. In particular, three types of particles were detected in the FESEM image which were related to the reacted gel phase, crystals, and unreacted fly ash particles. The unreacted fly ash particles remained present in the AAM samples even with the addition of BGWNP. In terms of crystal size, a crystalline structure size range of 150 to 300 nm was observed, as shown in Figure 11. In addition, the morphology of the crystals was needle-shaped, which were present around the fly ash particles. A similar observation was made by Jang et al. [91]. Moreover, it was found that some crystals were encapsulated by a gel product, which was distinguished by its dense layer. More non-reacted particles were observed when the addition level of BGWNP was set to higher than 10%. For example, Figure 11c,d present the morphology of the AAM incorporating 15% and 20% BGWNP, respectively. It was noticed that the structure of the AAM had a low density. From another point of view, Figure 12 presents the analysis results obtained from EDX test. Despite the important role of BGWNP in reducing the CaO content in the cement-based matrix, it was found that the calcium to silica ratio in the AAM containing 5% BGWNP was high (1.15). In contrast, the ratio of calcium to silicate in the AAM incorporating 15% was low (0.64). The increment of BGWNP content from 5% to 15% in AAM led to an increased ratio of SiO_2_ to Al_2_O_3_ from 1.74 to 2.55. This result confirmed that a higher amount of aluminum ions was substituted in the C,N-A-S-H chain. This result was in line with the experimental and predicted results of compressive strength, in which the increment of BGWNP content from 5% to 15% had significantly reduced the compressive strength owing to the limited amounts of Al_2_O_3_ and CaO and increasing amount of SiO_2_. This phenomenon led to a lower production of gels in comparison to the AAM containing 5% BGWNP.

## 4. Conclusions

This study investigated both experimental and informational modelling to optimise the mechanical properties of alkali-activated mortar made with different concentrations of FA, GBFS and BGWNP. According to the study outcome, the following conclusions could be drawn:

- BGWNP demonstrated its ability to improve the mechanical properties of AAM, however, the incorporation of BGWNP as the replacement of FA was found to be the most significant, while the replacement of GBFS by BGWNP was less significant.
- The compressive, tensile and flexural strength increased with an increase of BGWNP content as the replacement of FA by up to 10%. Nevertheless, the recorded optimum percentage was 8.4%, in which the values of compressive, tensile and flexural strength were 70.1, 4.67 and 7.59 MPa, respectively, which were higher than that of the control mixture (59.6, 3.8 and 6.5 MPa).
- The addition of BGWNP as the replacement of GBFS was limited up to 5% and the maximum compressive, tensile and flexural strength were 66.7, 4.2 and 7.2 MPa, respectively, at BGWNP of 3.4%. Beyond the said value, a significant strength reduction was observed.
- The insulation of BGWNP in AAM made with FA and GBFS (30:70) had a positive effect in which the compressive, tensile and flexural strength were 76.4, 4.5 and 7.4 MPa at an optimal BGWNP content of 6.9%.
- In general, the optimal replacement percentages of FA, GBFS, and BGWNP that maximise the compressive, tensile and flexural strength of AAM were 61.6%, 30%, and 8.4%, respectively.
- The non-linear equations proposed here proved their ability to predict the compressive, tensile and flexural strength with minimum error (RRMSE < 0.107, RRSE < 0.116 and RAE < 0.068) and high correlation between the actual and predicted data (*R*^2^ > 0.8601, *R* > 0.927), thus confirming both the robustness and reliability of the models.
- The differences between the adjusted *R*^2^ and predicted *R*^2^ were less than 0.2 for all equations, indicating that the models could be used for further observation in the future.

## Figures and Tables

**Table 1:** Chemical compositions of BGWNP, GBFS, and FA, mass %.

Composition	BGWNP	GBFS	FA
SiO_2_	69.14	30.7	57.25
Al_2_O_3_	13.86	11.1	28.75
Fe_2_O_3_	0.24	0.54	3.57
CaO	3.16	51.9	5.10
MgO	0.68	4.47	1.38
K_2_O	0.01	0.36	0.94
Na_2_O	0.01	0.45	0.09
SO_3_	4.08	0.06	0.10
LOI	0.16	0.22	0.11

**Table 2:** Max. and min. values of the independent variables.

Independent Variables	Code	Units	Level (−1)	Level (+1)
BGWNP	*X*_1_	%	0	20
BGWNP:GBFS	*X*_2_	%	0	20
BGWNP:FA	*X*_3_	%	0	20
Time	*X*_4_	days	7	90

**Table 3:** Predicted equations of compressive, tensile, and flexural strength for all models.

Model	Response	Predicted Equation
Model 1	Compressive strength (CS)	$CS=64.98-6.11X_{1}+13.11X_{4}-0.84X_{1}X_{4}-8.08X_{1}^{2}-15.72X_{4}^{2}$
	Flexural strength (FS)	$FS=7.32-0.48X_{1}+1.24X_{4}+0.065X_{1}X_{4}-0.8X_{1}^{2}-1.54X_{4}^{2}$
	Tensile strength (TS)	$TS=4.4-0.397X_{1}+0.614X_{4}-0.107X_{1}X_{4}-0.9X_{1}^{2}-0.65X_{4}^{2}$
Model 2	Compressive strength (CS)	$CS=62.01-8.43X_{1}+13.92X_{4}-1.53X_{1}X_{4}-7.06X_{1}^{2}-16.38X_{4}^{2}$
	Flexural strength (FS)	$FS=7.0-0.66X_{1}+1.35X_{4}-0.086X_{1}X_{4}-0.618X_{1}^{2}-1.77X_{4}^{2}$
	Tensile strength (TS)	$TS=3.97-0.5X_{1}+0.754X_{4}-0.146X_{1}X_{4}-0.43X_{1}^{2}-0.87X_{4}^{2}$
Model 3	Compressive strength (CS)	$CS=68.18-3.1X_{1}+13.64X_{4}-0.33X_{1}X_{4}-8.4X_{1}^{2}-16.15X_{4}^{2}$
	Flexural strength (FS)	$FS=7.47-0.259X_{1}+1.22X_{4}+0.075X_{1}X_{4}-0.79X_{1}^{2}-1.52X_{4}^{2}$
	Tensile strength (TS)	$TS=4.53-0.174X_{1}+0.839X_{4}-0.03X_{1}X_{4}-0.65X_{1}^{2}-0.93X_{4}^{2}$

**Table 4:** ANOVA results for the proposed models.

Item	Compressive Strength			Flexural Strength			Tensile Strength		
	*p*-Value	F-value	Sig.	*p*-Value	F-Value	Sig.	*p*-Value	F-Value	Sig.
**Model 1**	**<0.0001**	**17.22**	**Y**	**<0.0001**	**13.4**	**Y**	**0.0036**	**6.02**	**Y**
*X*_1_	0.0081	9.5		0.0375	5.28		0.0486	4.67	
*X*_4_	<0.0001	49.69		<0.0001	38.81		0.0031	12.67	
*X*_1_ *X*_4_	0.7526	0.103		0.8195	0.0541		0.6657	0.1948	
$X_{1}^{2}$	0.0295	5.88		0.0402	5.11		0.0109	8.6	
$X_{4}^{2}$	0.0002	24.24		0.0005	20.17		0.0453	4.83	
**Model 2**	**<0.0001**	**20.01**	**Y**	**<0.0001**	**18.13**	**Y**	**<0.0001**	**15.08**	**Y**
*X*_2_	0.0008	17.85		0.0056	10.68		0.0015	15.49	
*X*_4_	<0.0001	55.24		<0.0001	50.71		<0.0001	39.87	
*X*_2_ *X*_4_	0.5722	0.3345		0.7524	0.1035		0.3985	0.7585	
$X_{2}^{2}$	0.0538	4.43		0.0902	3.31		0.0630	4.08	
$X_{4}^{2}$	0.0002	25.98		<0.0001	29.49		0.0008	18.22	
**Model 3**	**<0.0001**	**18.65**	**Y**	**<0.0001**	**13.91**	**Y**	**<0.0001**	**19.39**	**Y**
*X*_3_	0.1232	2.69		0.216	1.68		0.1505	2.31	
*X*_4_	<0.0001	59.18		<0.0001	42.55		<0.0001	61.14	
*X*_3_ *X*_4_	0.8966	0.0175		0.7803	0.0808		0.8319	0.0468	
$X_{3}^{2}$	0.0193	6.99		0.0329	5.6		0.0043	11.59	
$X_{4}^{2}$	0.0001	28.16		0.0003	22.4		0.0002	25.53	

**Table 5:** Statistical validation methods for the compressive strength of all models.

Item	*R*	RRMSE	RMSE	RRSE	RSE	RAE	MAE
Model 1	0.860	0.102	5.218	0.114	0.012	0.063	4.165
Model 2	0.877	0.107	5.253	0.116	0.013	0.068	4.411
Model 3	0.870	0.0925	4.973	0.106	0.011	0.061	4.17

## Data Availability

The data presented in this study are available on request from the corresponding author.
